# *N*-[2-(2-Chloro­phen­yl)-2-hydroxy­ethyl]propan-2-aminium chloride

**DOI:** 10.1107/S1600536809031146

**Published:** 2009-08-19

**Authors:** Bi-Wei Song, Lin-Jun Xie, Ling-Ling Dong, Zhan Tang, Hai Feng

**Affiliations:** a College of Pharmaceutical Sciences, Zhejiang University of Technology, Hangzhou 310014, People’s Republic of China; b College of Mechanical Engineering, Zhejiang University of Technology, Hangzhou 310014, People’s Republic of China

## Abstract

In the title compound, C_11_H_17_ClNO^+^·Cl^−^, the side chain of the ethyl­amine group is orientated approximately perpendicular to the benzene ring, the dihedral angle between the C/C/N plane of the ethyl­amine group and the benzene plane being 83.5 (3)°. In the crystal structure, inter­molecular O—H⋯Cl and N—H⋯Cl hydrogen bonds are observed. The crystal studied was an inversion twin with a 0.51 (10):0.49 (10) domain ratio.

## Related literature

For a related structure, see: Tang *et al.* (2009 ▶).
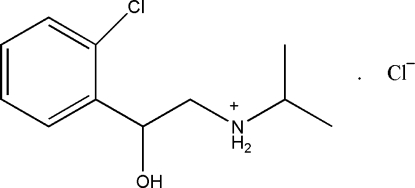


## Experimental

### 

#### Crystal data

C_11_H_17_ClNO^+^·Cl^−^
*M* *_r_* = 250.16
Orthorhombic, 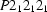

*a* = 7.3460 (3) Å
*b* = 11.7721 (5) Å
*c* = 15.2377 (8) Å
*V* = 1317.72 (10) Å^3^
*Z* = 4
Mo *K*α radiation
μ = 0.47 mm^−1^
*T* = 296 K
0.40 × 0.36 × 0.32 mm

#### Data collection

Rigaku R-AXIS RAPID diffractometer
Absorption correction: multi-scan (**ABSCOR**; Higashi, 1995 ▶) *T*_min_ = 0.835, *T*_max_ = 0.864
12577 measured reflections
2977 independent reflections
1874 reflections with *I* > 2σ(*I*)
*R*_int_ = 0.031

#### Refinement

*R*[*F* ^2^ > 2σ(*F* ^2^)] = 0.032
*wR*(*F* ^2^) = 0.107
*S* = 1.00
2977 reflections
140 parameters
H-atom parameters constrained
Δρ_max_ = 0.29 e Å^−3^
Δρ_min_ = −0.29 e Å^−3^
Absolute structure: Flack (1983 ▶), 1243 Friedel pairs
Flack parameter: 0.51 (10)

### 

Data collection: *PROCESS-AUTO* (Rigaku, 2006 ▶); cell refinement: *PROCESS-AUTO*; data reduction: *CrystalStructure* (Rigaku/MSC, 2007 ▶); program(s) used to solve structure: *SHELXS97* (Sheldrick, 2008 ▶); program(s) used to refine structure: *SHELXL97* (Sheldrick, 2008 ▶); molecular graphics: *ORTEP-3 for Windows* (Farrugia, 1997 ▶); software used to prepare material for publication: *WinGX* (Farrugia, 1999 ▶).

## Supplementary Material

Crystal structure: contains datablocks global, I. DOI: 10.1107/S1600536809031146/is2447sup1.cif

Structure factors: contains datablocks I. DOI: 10.1107/S1600536809031146/is2447Isup2.hkl

Additional supplementary materials:  crystallographic information; 3D view; checkCIF report

## Figures and Tables

**Table 1:** Hydrogen-bond geometry (Å, °)

*D*—H⋯*A*	*D*—H	H⋯*A*	*D*⋯*A*	*D*—H⋯*A*
N1—H112⋯Cl2	0.90	2.36	3.199 (2)	156
O1—H1⋯Cl2^i^	0.82	2.33	3.143 (2)	169
N1—H111⋯Cl2^ii^	0.90	2.28	3.138 (2)	160

Symmetry codes: (i) 
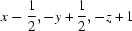
; (ii) 
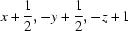
.

**Fig. 1.**
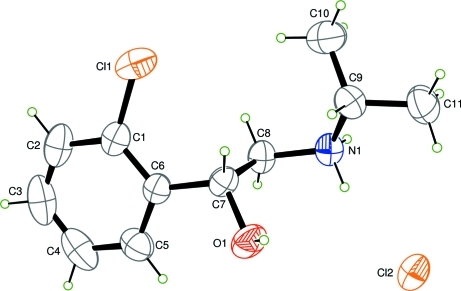
The molecular structure of (I) with atom labels, showing 40% probability displacement ellipsoids.

## supplementary crystallographic information

### Comment

The title compound (clorprenaline hydrochloride) is one of a series of structurally related β-adrenoceptorblocking drugs.

In the molecular structure (Fig. 1), there are no unusual bond distances or angles. The Cl atom and the phenyl plane is almost planar with the deviation of 0.0037 Å. The dihedral angle between the plane formed by C7/C8/N1 and the phenyl plane is 83.5 (3)°, which shows that the two planes are almost perpendicular. The C9—N1 distance of 1.506 Å is longer than the value of the similar bond distance of 1.474 Å (Tang *et al.*, 2009).

O—H···Cl and N—H···Cl hydrogen bonds are found in the crystal structure and are essential forces in crystal formation. The hydroxyl hydrogen at O1 acts as a donor to Cl2. The ethylamine hydrogens at N1 also act as donors to Cl2.

### Experimental

Racemic Clorprenaline hydrochloride was purchased from ShangHai Shengxin Medicine & Chemical Co., Ltd. ShangHai, China. Racemic Clorprenaline hydrochloride (5 g) was dissolved in ethanol (75 ml) and then hydrochloric acid was added to give pH of about 4. Colorless crystal of (I) separated from the solution in about 80% yield after one day.

### Refinement

All of the H atoms were placed in calculated positions and allowed to ride on their parent atoms, with C—H = 0.93 (aromatic), 0.98 (methine), 0.97 (methylene), 0.96 Å (methyl), O—H = 0.82 Å and N—H = 0.90 Å, and with *U*_iso_(H) = 1.2–1.5 times *U*_eq_ of the parent atoms.

### Crystal data

**Table:**

C_11_H_17_ClNO^+^·Cl^−^	*F*(000) = 528
*M**_r_* = 250.16	*D*_x_ = 1.261 Mg m^−^^3^
Orthorhombic, *P*2_1_2_1_2_1_	Mo *K*α radiation, λ = 0.71073 Å
Hall symbol: P 2ac 2ab	Cell parameters from 8627 reflections
*a* = 7.3460 (3) Å	θ = 3.1–27.4°
*b* = 11.7721 (5) Å	µ = 0.47 mm^−^^1^
*c* = 15.2377 (8) Å	*T* = 296 K
*V* = 1317.72 (10) Å^3^	Chunk, colorless
*Z* = 4	0.40 × 0.36 × 0.32 mm

### Data collection

**Table:**

Rigaku R-AXIS RAPID diffractometer	2977 independent reflections
Radiation source: rotating anode	1874 reflections with *I* > 2σ(*I*)
graphite	*R*_int_ = 0.031
Detector resolution: 10.00 pixels mm^-1^	θ_max_ = 27.4°, θ_min_ = 3.1°
ω scans	*h* = −9→9
Absorption correction: multi-scan (*ABSCOR*; Higashi, 1995)	*k* = −15→14
*T*_min_ = 0.835, *T*_max_ = 0.864	*l* = −19→19
12577 measured reflections	

### Refinement

**Table:**

Refinement on *F*^2^	Hydrogen site location: inferred from neighbouring sites
Least-squares matrix: full	H-atom parameters constrained
*R*[*F*^2^ > 2σ(*F*^2^)] = 0.032	*w* = 1/[σ^2^(*F*_o_^2^) + (0.0431*P*)^2^ + 0.5*P*] where *P* = (*F*_o_^2^ + 2*F*_c_^2^)/3
*wR*(*F*^2^) = 0.107	(Δ/σ)_max_ < 0.001
*S* = 1.00	Δρ_max_ = 0.29 e Å^−^^3^
2977 reflections	Δρ_min_ = −0.29 e Å^−^^3^
140 parameters	Extinction correction: *SHELXL97* (Sheldrick, 2008), Fc^*^=kFc[1+0.001xFc^2^λ^3^/sin(2θ)]^-1/4^
0 restraints	Extinction coefficient: 0.0054 (12)
Primary atom site location: structure-invariant direct methods	Absolute structure: Flack (1983), 1243 Friedel pairs
Secondary atom site location: difference Fourier map	Flack parameter: 0.51 (10)

### Special details

**Table:**

Geometry. All e.s.d.'s (except the e.s.d. in the dihedral angle between two l.s. planes) are estimated using the full covariance matrix. The cell e.s.d.'s are taken into account individually in the estimation of e.s.d.'s in distances, angles and torsion angles; correlations between e.s.d.'s in cell parameters are only used when they are defined by crystal symmetry. An approximate (isotropic) treatment of cell e.s.d.'s is used for estimating e.s.d.'s involving l.s. planes.
Refinement. Refinement of *F*^2^ against ALL reflections. The weighted *R*-factor *wR* and goodness of fit *S* are based on *F*^2^, conventional *R*-factors *R* are based on *F*, with *F* set to zero for negative *F*^2^. The threshold expression of *F*^2^ > σ(*F*^2^) is used only for calculating *R*-factors(gt) *etc*. and is not relevant to the choice of reflections for refinement. *R*-factors based on *F*^2^ are statistically about twice as large as those based on *F*, and *R*- factors based on ALL data will be even larger.

### Fractional atomic coordinates and isotropic or equivalent isotropic displacement parameters (Å^2^)

**Table:**

	*x*	*y*	*z*	*U*_iso_*/*U*_eq_	
Cl2	0.51205 (10)	0.14865 (6)	0.44638 (6)	0.0648 (2)	
Cl1	0.44580 (11)	0.75320 (8)	0.58138 (6)	0.0779 (3)	
O1	0.3177 (3)	0.44304 (18)	0.42787 (16)	0.0698 (6)	
H1	0.2294	0.4174	0.4542	0.105*	
N1	0.5869 (3)	0.37691 (19)	0.55437 (15)	0.0522 (6)	
H111	0.7029	0.3550	0.5628	0.063*	
H112	0.5341	0.3244	0.5198	0.063*	
C7	0.4020 (4)	0.5277 (2)	0.4795 (2)	0.0489 (7)	
H7	0.3296	0.5422	0.5323	0.059*	
C6	0.4249 (3)	0.6368 (2)	0.42750 (19)	0.0488 (6)	
C1	0.4496 (3)	0.7418 (2)	0.4678 (2)	0.0546 (7)	
C8	0.5901 (4)	0.4868 (2)	0.5047 (2)	0.0537 (7)	
H8A	0.6623	0.4771	0.4519	0.064*	
H8B	0.6487	0.5443	0.5405	0.064*	
C9	0.4922 (4)	0.3739 (2)	0.64202 (18)	0.0579 (7)	
H9	0.3639	0.3936	0.6332	0.069*	
C5	0.4305 (4)	0.6352 (3)	0.3365 (2)	0.0680 (9)	
H5	0.4144	0.5669	0.3069	0.082*	
C11	0.5028 (6)	0.2537 (3)	0.6769 (2)	0.0777 (9)	
H11A	0.6279	0.2330	0.6854	0.093*	
H11B	0.4477	0.2027	0.6355	0.093*	
H11C	0.4393	0.2492	0.7319	0.093*	
C2	0.4764 (4)	0.8401 (3)	0.4204 (3)	0.0729 (10)	
H2	0.4904	0.9091	0.4494	0.088*	
C3	0.4824 (5)	0.8360 (4)	0.3318 (3)	0.0920 (13)	
H3	0.5018	0.9022	0.2998	0.110*	
C4	0.4597 (5)	0.7344 (5)	0.2889 (3)	0.0897 (12)	
H4	0.4639	0.7319	0.2279	0.108*	
C10	0.5746 (6)	0.4584 (3)	0.7044 (2)	0.0928 (13)	
H10A	0.5099	0.4564	0.7591	0.111*	
H10B	0.5665	0.5332	0.6797	0.111*	
H10C	0.7001	0.4396	0.7144	0.111*	

### Atomic displacement parameters (Å^2^)

**Table:**

	*U*^11^	*U*^22^	*U*^33^	*U*^12^	*U*^13^	*U*^23^
Cl2	0.0489 (4)	0.0509 (3)	0.0944 (6)	0.0007 (3)	0.0009 (4)	−0.0054 (4)
Cl1	0.0692 (5)	0.0819 (6)	0.0825 (6)	−0.0038 (5)	−0.0021 (4)	−0.0313 (5)
O1	0.0628 (13)	0.0559 (12)	0.0909 (18)	−0.0136 (10)	−0.0123 (12)	−0.0113 (12)
N1	0.0435 (11)	0.0498 (13)	0.0633 (15)	−0.0017 (10)	−0.0009 (11)	0.0057 (11)
C7	0.0399 (13)	0.0455 (14)	0.0613 (17)	−0.0049 (12)	−0.0016 (12)	−0.0044 (13)
C6	0.0393 (12)	0.0465 (14)	0.0606 (18)	0.0017 (12)	−0.0015 (12)	0.0003 (13)
C1	0.0364 (13)	0.0510 (15)	0.0765 (19)	0.0010 (13)	0.0021 (13)	−0.0009 (15)
C8	0.0438 (14)	0.0483 (15)	0.069 (2)	−0.0023 (13)	0.0006 (13)	0.0073 (13)
C9	0.0513 (15)	0.0647 (18)	0.0577 (17)	−0.0011 (16)	0.0030 (15)	0.0076 (13)
C5	0.0671 (19)	0.080 (2)	0.057 (2)	0.0136 (19)	0.0015 (15)	0.0010 (17)
C11	0.087 (2)	0.074 (2)	0.071 (2)	−0.006 (3)	0.003 (2)	0.0193 (17)
C2	0.0455 (15)	0.0489 (16)	0.124 (3)	−0.0015 (15)	0.007 (2)	0.0108 (18)
C3	0.059 (2)	0.082 (3)	0.135 (4)	0.012 (2)	0.010 (2)	0.050 (3)
C4	0.076 (3)	0.125 (3)	0.068 (2)	0.020 (3)	0.0058 (19)	0.039 (2)
C10	0.123 (3)	0.086 (3)	0.069 (2)	−0.011 (3)	−0.002 (2)	−0.012 (2)

### Geometric parameters (Å, °)

**Table:**

Cl1—C1	1.736 (3)	C9—C11	1.514 (4)
O1—C7	1.413 (3)	C9—H9	0.9800
O1—H1	0.8200	C5—C4	1.391 (5)
N1—C8	1.499 (3)	C5—H5	0.9300
N1—C9	1.506 (3)	C11—H11A	0.9600
N1—H111	0.9000	C11—H11B	0.9600
N1—H112	0.9000	C11—H11C	0.9600
C7—C8	1.513 (4)	C2—C3	1.352 (6)
C7—C6	1.518 (4)	C2—H2	0.9300
C7—H7	0.9800	C3—C4	1.373 (7)
C6—C5	1.387 (4)	C3—H3	0.9300
C6—C1	1.393 (4)	C4—H4	0.9300
C1—C2	1.378 (5)	C10—H10A	0.9600
C8—H8A	0.9700	C10—H10B	0.9600
C8—H8B	0.9700	C10—H10C	0.9600
C9—C10	1.503 (5)		
			
C7—O1—H1	109.5	C10—C9—H9	108.5
C8—N1—C9	118.4 (2)	N1—C9—H9	108.5
C8—N1—H111	107.7	C11—C9—H9	108.5
C9—N1—H111	107.7	C6—C5—C4	121.0 (4)
C8—N1—H112	107.7	C6—C5—H5	119.5
C9—N1—H112	107.7	C4—C5—H5	119.5
H111—N1—H112	107.1	C9—C11—H11A	109.5
O1—C7—C8	108.5 (2)	C9—C11—H11B	109.5
O1—C7—C6	110.8 (2)	H11A—C11—H11B	109.5
C8—C7—C6	107.5 (2)	C9—C11—H11C	109.5
O1—C7—H7	110.0	H11A—C11—H11C	109.5
C8—C7—H7	110.0	H11B—C11—H11C	109.5
C6—C7—H7	110.0	C3—C2—C1	119.9 (3)
C5—C6—C1	116.7 (3)	C3—C2—H2	120.1
C5—C6—C7	120.9 (3)	C1—C2—H2	120.1
C1—C6—C7	122.4 (2)	C2—C3—C4	120.2 (3)
C2—C1—C6	122.2 (3)	C2—C3—H3	119.9
C2—C1—Cl1	117.4 (3)	C4—C3—H3	119.9
C6—C1—Cl1	120.4 (2)	C3—C4—C5	120.1 (4)
N1—C8—C7	112.9 (2)	C3—C4—H4	120.0
N1—C8—H8A	109.0	C5—C4—H4	120.0
C7—C8—H8A	109.0	C9—C10—H10A	109.5
N1—C8—H8B	109.0	C9—C10—H10B	109.5
C7—C8—H8B	109.0	H10A—C10—H10B	109.5
H8A—C8—H8B	107.8	C9—C10—H10C	109.5
C10—C9—N1	111.1 (3)	H10A—C10—H10C	109.5
C10—C9—C11	112.1 (3)	H10B—C10—H10C	109.5
N1—C9—C11	108.0 (2)		
			
O1—C7—C6—C5	−23.6 (4)	C6—C7—C8—N1	−178.2 (2)
C8—C7—C6—C5	94.8 (3)	C8—N1—C9—C10	−58.5 (3)
O1—C7—C6—C1	159.4 (2)	C8—N1—C9—C11	178.2 (3)
C8—C7—C6—C1	−82.2 (3)	C1—C6—C5—C4	0.1 (4)
C5—C6—C1—C2	0.6 (4)	C7—C6—C5—C4	−177.1 (3)
C7—C6—C1—C2	177.8 (2)	C6—C1—C2—C3	−1.1 (4)
C5—C6—C1—Cl1	−179.8 (2)	Cl1—C1—C2—C3	179.4 (3)
C7—C6—C1—Cl1	−2.7 (3)	C1—C2—C3—C4	0.7 (5)
C9—N1—C8—C7	−62.6 (3)	C2—C3—C4—C5	0.0 (6)
O1—C7—C8—N1	−58.4 (3)	C6—C5—C4—C3	−0.5 (5)

### Hydrogen-bond geometry (Å, °)

**Table:**

*D*—H···*A*	*D*—H	H···*A*	*D*···*A*	*D*—H···*A*
N1—H112···Cl2	0.90	2.36	3.199 (2)	156
O1—H1···Cl2^i^	0.82	2.33	3.143 (2)	169
N1—H111···Cl2^ii^	0.90	2.28	3.138 (2)	160

Symmetry codes: (i) *x*−1/2, −*y*+1/2, −*z*+1; (ii) *x*+1/2, −*y*+1/2, −*z*+1.
